# Robotic video-assisted thoracoscopic surgery using multiport triangular trocar configuration: initial experience at a single center

**DOI:** 10.1186/s13019-021-01455-5

**Published:** 2021-04-13

**Authors:** Vu Huu Vinh, Nguyen Viet Dang Quang, Dang Dinh Minh Thanh, Truong Van Le Phong

**Affiliations:** Department of Thoracic Surgery, Choray Hospital, 202B Nguyen Chi Thanh Street, District No. 5, Hochiminh City, Vietnam

**Keywords:** Conventional, Outcomes, Robotic video-assisted thoracoscopic surgery, Triangular, Trocar

## Abstract

**Background:**

Recent developments in robotic technology have brought significant changes in robotic video-assisted thoracoscopic surgery (r-VATS) worldwide, particularly including the treatment in the thorax for the mediastinal, esophagus, and pulmonary lesions. Currently, there are only a few reports describing the procedural experience and outcomes with r-VATS. The objective of this study is to provide our initial experience using r-VATS at a single center, with specific attention to safety, efficacy, and procedural details.

**Methods:**

We retrospectively reviewed patients who underwent a newly modified r-VATS procedure for various surgical operations at the thoracic department of our hospital, from July 2018 to January 2020. Multiport trocars were placed in the classic triangular arrangement as in conventional VATS (c-VATS) but with modifications based on the type of surgery. The peri- and postoperative outcomes such as duration of surgery, complications, and duration of hospital stay for these patients were reported.

**Results:**

Overall, 142 patients underwent r-VATS for lobectomy (66), wedge resection (15), thymectomy (22), mediastinal tumor resection (30), pneumonectomy (4), transthoracic esophagectomy (1), esophageal tumor resection or esophageal diverticulum repair (2), diaphragm plication (1), and mediastinal tumor resection plus thymectomy (1). For the entire cohort, the median operative time was 110 min, and the median length of hospital stay was 5 days. Conversion to open thoracic surgery was reported only in a total of 3 (2.1%) patients of pneumonectomy (1.4%) and mediastinal tumor resection (0.70%). All our patients were managed successfully with no postoperative complications and mortality.

**Conclusion:**

Our method of r-VATS was found to be safe and effective and may be applied to different surgical operations. Adequate and proper training of thoracic surgeons is immediately needed for the transition from c-VATS to r-VATS**.** The utility and advantages of triangular trocar configuration for r-VATS require further refinement and research before it can be routinely adopted in clinical practice.

**Trial registration:**

Retrospectively registered.

## Introduction

Even though there are few concerns about the safety of video-assisted thoracoscopic surgery (VATS), this minimally invasive approach has several advantages over traditional open thoracic surgery for performing lobectomies, wedge resections, and segmentectomies. These advantages include less bleeding during the surgery, lesser postoperative pain, smaller surgical incisions reducing the exposure to internal organs, shorter hospitalization time, fewer postoperative complications, and shorter recovery times [1–3]. However, the usage of VATS is restricted due to challenging technicalities of the procedures viz. requirement of a high level of thoracoscopic skills by the surgeon, low flexibility of instruments, limited surgical space, and poor 2-dimensional visualization of the video camera [4]. Currently, the interest in surgical procedures using a robot-assisted device is evolving. In the 1980s, after the introduction of robot-assisted surgery [5], the first telerobotic surgery was documented in 2002 in a patient undergoing cholecystectomy [6]. Since then, robotic surgeries have been applied to different specialties, and robotic thoracoscopic surgery (RATS) has been adopted by a large number of thoracic surgeons across the world.

By including a robot-assisted surgical system to VATS, several drawbacks of video-thoracoscopic surgical cameras and instruments are addressed as the robot arms exhibit greater precision, improved dexterity due to superior range of motion, and also provide a high definition 3-dimensional view of the operating field adding to the comfort of the surgeon [7–9]. At present, there are only a few reports describing the efficiency of procedures involving robotic assistance for VATS [10]. We, therefore, performed a retrospective analysis to evaluate the peri-and postoperative outcomes of a modified robotic VATS (r-VATS) technique employing triangular trocar placement [as in conventional VATS (c-VATS)] in patients undergoing different types of thoracoscopic surgeries at our hospital.

## Methods

This study was a retrospective analysis of data collected from July 2018 to Jan 2020. It included patients who underwent r-VATS for different surgical operations such as lobectomy, wedge resection, thymectomy, mediastinal tumor resection, pneumonectomy, transthoracic esophagectomy, esophageal tumor resection or esophageal diverticulum repair, diaphragm plication, and mediastinal tumor resection plus thymectomy at the thoracic department of our hospital. The inclusion criteria for this study was all the patients with indication of thoracic surgery while the exclusion criteria were (1) patients with large size of tumor, severely invading to adjacent great vessels or the heart (2) patients with severe comorbidity and not be able to withstand the longer duration of anesthesia during robotic surgery. Peri- and postoperative outcomes such as operative time, adverse events or complications, and duration of hospital stay for all the patients were recorded. At the time of admission, written informed consent was obtained from all participating patients. All the study procedures were conducted in accordance with the Declaration of Helsinki and the Institutional Ethics Committee.

### Surgical procedure

**Fig. 1:**
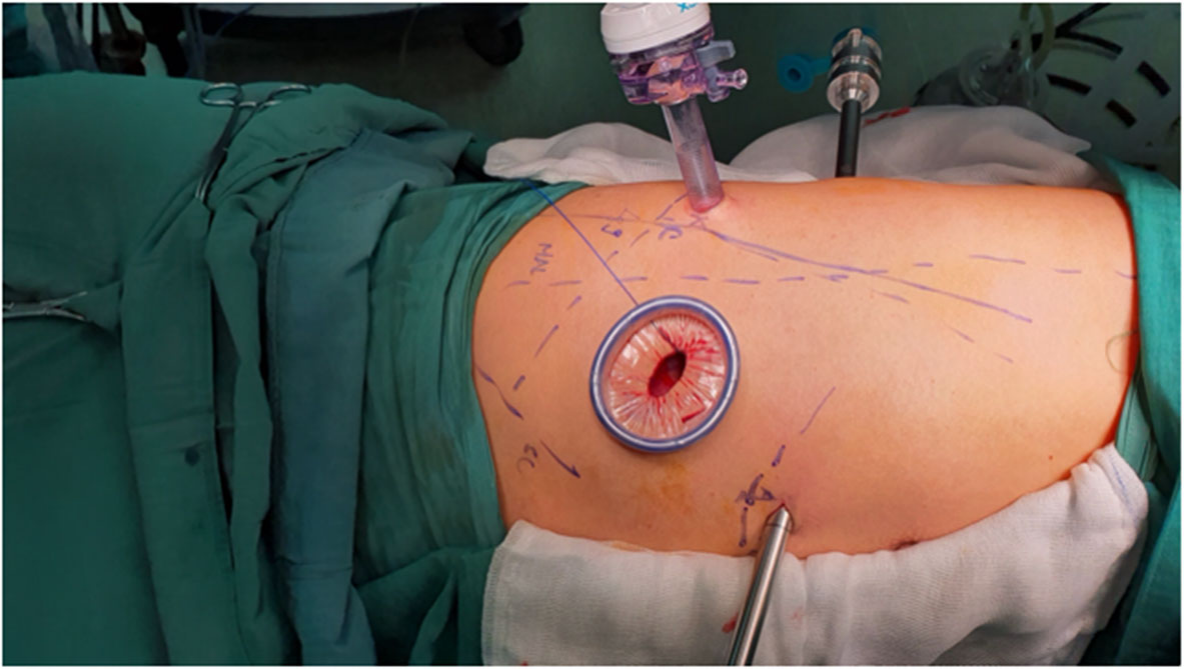
The trocars and working incision (with a wound retractor) in a patient

For this study, a newly modified procedure for r-VATS was evaluated. The robotic platform used for surgeries belonged to the third-generation Si™ system by da Vinci, USA (Intuitive Surgicals), with four patient cart arms. These robotic arms enable the surgeon, sitting in his console to maneuver endoscope and other instruments in the surgical site. We used four different trocars or ports; two (each 8 mm) for two instrument arms (arms 1 and 2), one for the camera (12 mm; arm 3), and a 1.5 cm working incision with a wound retractor for assistant works (Fig. 1). These trocars were positioned in the classic endoscopic triangular configuration similar to that in our multiport conventional VATS (c-VATS) procedure, but with modifications based on the type of surgeries as described below.

**Fig. 2:**
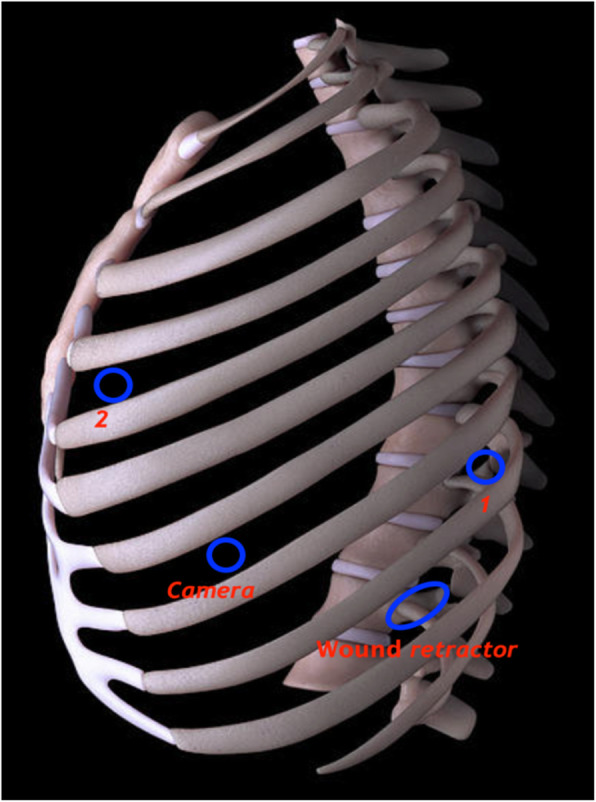
The placement of multiport trocars in a triangular configuration in the left side for operations in the lateral thoracic cavities such as lobectomy, esophagectomy, esophageal cyst, or muscle benign tumor resection, or posterior mediastinal tumor resection. 1,2: robotic arms 1 and 2; wound retractor: 1.5 cm assistant port, acting as a working incision; camera: camera port

**Fig. 3:**
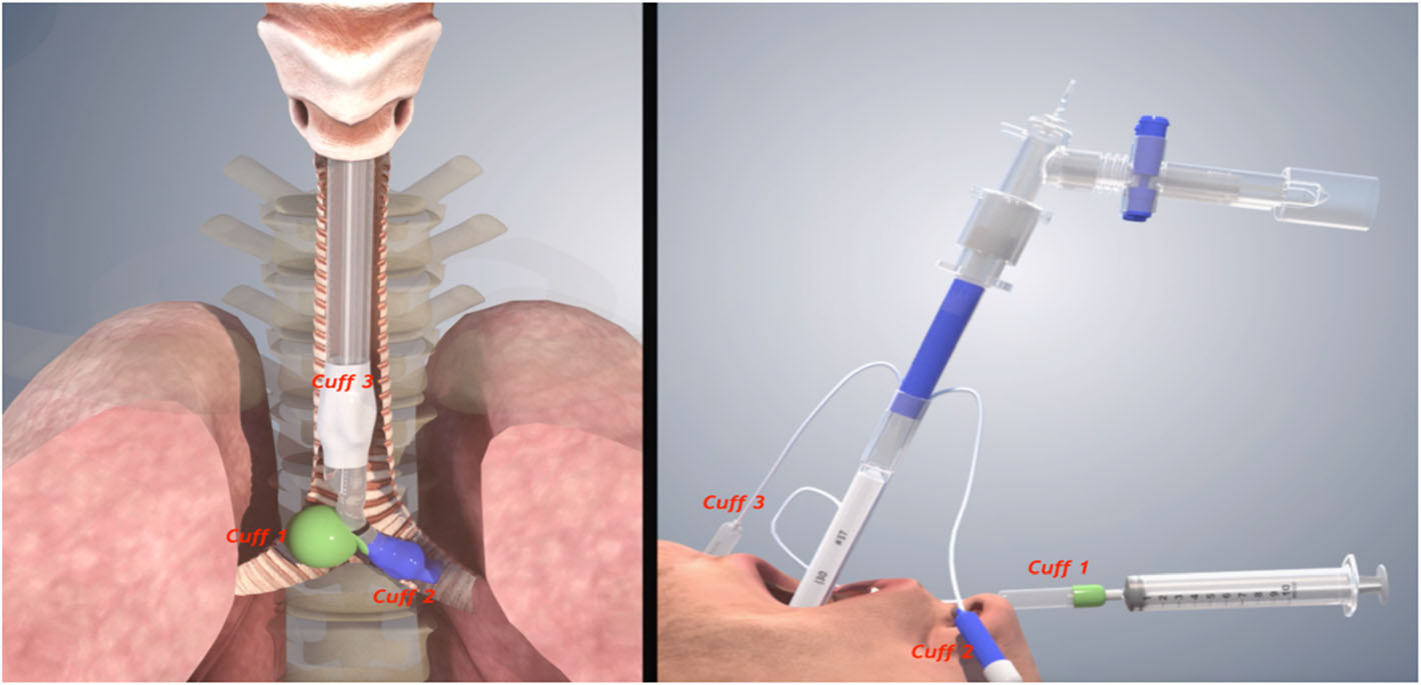
Picture depicting a 3-cuffed endobronchial double-lumen tube intubation and its mechanical work

For operations in the lateral thoracic cavities such as lobectomy, esophagectomy, esophageal cyst or muscle benign tumor resection, or posterior mediastinal tumor resection, the patients were placed in a lateral decubitus position under general anesthesia. The first and second trocar incisions were placed in the 8th and 4th intercostal space (ICS), respectively. An additional trocar for the camera was placed in the 7th ICS. The 4th trocar incision was placed in the 9th ICS (Fig. 2). This 4th port acted as a working incision as in c-VATS, and from this port, the assistant surgeon could assist the main surgeon (sitting outside the sterile field) in various processes such as sucking, retracting, stapling or taking out surgical material and manipulating surgical firing during the operation. After the surgery, the assistant working incision with a size of 1.5 cm was good enough to remove the specimen even with a big tumor, without requiring further incisions. Using the working incision as an assistant port, however, kept the chest cavity exposed to the atmospheric air. Therefore, CO_2_ insufflation could not be used, and the affected lung was deflated by one-lung ventilation. For better deflation of the affected lung, a 3-cuffed endobronchial double-lumen tube (Ankor®, Korea) was used. The 3rd additional cuff is to help the bronchial tip to sit exactly in the desired position in the opposite main bronchus (Fig. 3).

**Fig. 4:**
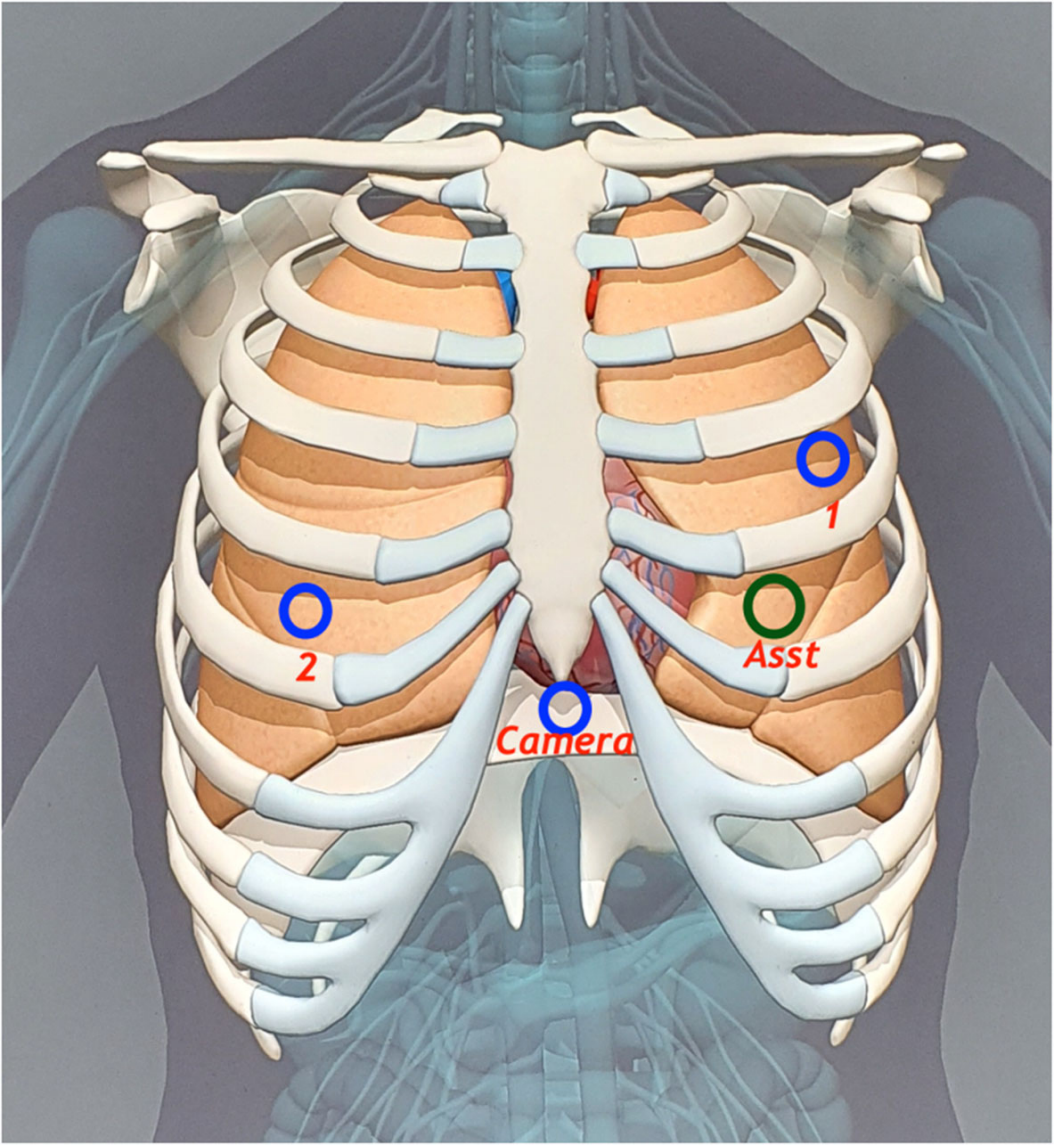
The placement of multiport trocars in a triangular configuration. For surgeries in the anterior mediastinum in the supine position, while the trocar for the camera was placed just below the sternum xiphoid, trocars for arm 1, 2, and assistant were put bilaterally. 1, 2: robotic arms 1 and 2 (each 8 mm); Asst: assistant port or trocar, valved at 12 mm, assistant surgeon use this port to assist during the operation; camera: camera port

For operations in the anterior mediastinum, such as anterior mediastinal tumor resection, thymectomy (including with and without thymoma), patients were put down in supine position with subxiphoid camera port and bilaterally placed incisions for robot arms (1 and 2) and assistant port (Fig. 4). In these cases, CO_2_ insufflation was used to create more room for surgical manipulation. Therefore, the assistant port was a valved trocar (12 mm) and not a working incision with wound retractor, as in the case of operations in the lateral thoracic cavities.

### Statistical analysis

All statistical analyses were performed using GraphPad InStat software version 3. Descriptive statistics were used to report the means, medians, and standard deviations for the continuous study variables and the number and percent for categoric variables.

## Results

### Baseline demographic data

A total of 142 eligible patients who satisfied the inclusion/exclusion criteria were enrolled in the study. The total number of males and females included in the study was 85(60%) and 57(40%), respectively. The mean age of the patients was 54.35 ± 14.39 years (Mean ± SD). The baseline demographic characteristics of these patients, stratified on the basis of the type of surgical operation performed by r-VATS, have been provided in Table 1.

**Table 1:** Patient demographic characteristics stratified by the type of surgical operation performed by r-VATS

S.No	Type of surgical operations	Cases (***n*** = 142)	Patient characteristics	
			Mean Age (years)	Gender (Male/Female; n)
			(Mean ± SD)	
1	Lobectomy	66	60.67 **±** 8.20	Male = 47(71.21%)
				Female = 19(28.79%)
2	Wedge resection	15	60.60 **±** 11.89	Male = 13(86.67%)
				Female = 2(13.33%)
3	Thymectomy	22	40.27 **±** 14.55	Male = 6(27.27%)
				Female = 16(72.73%)
4	Mediastinal tumor resection	30	47.43 **±** 16.35	Male = 12(40%)
				Female = 18(60%)
5	Pneumonectomy	4	59 **±** 6.38	Male = 4(100%)
6	Transthoracic esophagectomy	1	54	Male = 1(100%)
7	Esophageal tumor resection/ Esophageal diverticulum repair	2	53 **±** 32.53	Male = 1(50%)
				Female = 1(50%)
8	Diaphragm plication	1	61	Male = 1(100%)
9	Mediastinal tumor resection plus thymectomy	1	40	Female = 1(100%)

### Peri- and postoperative outcomes

Median operative time for the entire cohort undergoing r-VATS procedure was 110 (60–280) minutes. The overall conversion rate to open thoracic surgery was 2.1% (3 cases); 1.4% for pneumonectomy (2 cases) and 0.70% for mediastinal tumor resection (1 case). The median length of hospital stay for the cohort was 4.5 (3–12) days. None of the patients experienced any postoperative complications. No operative mortality for patients was reported. The details of peri- and postoperative outcomes for each type of surgery have been provided in Table 2.

**Table 2:** Peri- and postoperative outcomes of r-VATS for different surgical operations

Type of surgical operations	No. of patients (n)	Diagnosis	Pathology (n)	Median operative time (range); in minutes	Conversion rate for each surgery (%)	Median duration of hospital stay (range); in days
Lobectomy	66	Tumors in the right upper or lower lobes (RUL/RLL) and the left upper or lower lobes (LUL/LLL) of the lungs/ LLL bronchiectasis	Malignant adenocarcinoma (63) and malignant squamous cell carcinoma (3)	120 (80–220)	NA	5 (3–16)
Wedge resection	15	LUL/RUL/LLL/RLL/LU + LL tumor	Benign inflammation (13) and tuberculosis (2)	60 (50–80)	NA	3 (2–4)
Thymectomy	22	Thymoma/Myasthenia gravis/ Thymoma+Myasthenia gravis	Benign myasthenia (19) and myasthenia +small thymoma (3)	80 (60–180)	NA	4 (3–10)
Mediastinal tumor resection	30	Mediastinal tumor	Posterior benign neurogenic tumors (9), posterior paratracheal benign cyst (2), invasive thymoma (12) thymic carcinomas (7; one converted to open)	110 (60–190)	3.33	4 (3–8)
Pneumonectomy	4	LLL/LUL/RLL tumor	Malignant adenocarcinoma (4)	157.50 (95–220)	50	6.5 (4–10)
Transthoracic esophagectomy	1	Esophageal tumor	Malignant squamous cell carcinoma (1)	280	NA	12
Esophageal tumor resection/ Esophageal diverticulum repair	2	Esophageal leiomyoma	Benign (2)	115 (110–120)	NA	4.5 (4–5)
Diaphragm plication	1	Elevated diaphragm	NA	70	NA	3
Mediastinal tumor resection plus thymectomy	1	Thymoma	Benign (1)	100	NA	6

## Discussion

Robotic surgery is considered as the future of surgery amongst the entire medical fraternity; owing to its rapid development, easy adaptations, and the impact that it has made to existing laparoscopic procedures in the last two decades.

The driving force that ultimately led to the developments in the field of laparoscopy was derived from the collaboration between NASA’s Ames Research Centre and researchers from Stanford and was based on the concept of telerobotic surgery. In 1990, this idea got commercialized, and Computer Motion, USA, designed and developed a robotic platform called the Automated Endoscopic System for Optimal Positioning (AESOP), which combined the telemanipulator with a foot pedal [11, 12]. Further modifications in the system led to the launch of the Zeus operating system in the markets in 1998 that was originally designed for cardiac surgery but later was found extending to other surgeries as well [12–15]. Around the same time, in the late 1990s the da Vinci Surgical System (Intuitive Surgical, Sunnyvale, California) was introduced and in 2000 it became the first robotic surgical system to be approved by the FDA for general laparoscopic surgery (i.e., for gallbladder disease and gastroesophageal reflux). It is the only robotic surgical system to be used nowadays around the globe and represents a 3–4 armed system with a central endoscope holding a binocular lens providing a 3-dimensional (3D) view of the surgical field. However, the most striking feature of this surgical system is the EndoWrist technology, capable of 7 degrees of freedom, thus replicating the mobility like that of a human hand [10, 16]. This allows surgeons to perform complex minimally invasive surgical procedures with high precision and accuracy. Robotic surgery has thus made spectacular progress in handling even difficult situations related to manipulating blood vessels that are most vulnerable in converting an endoscopic surgery to an open one.

Robotically assisted surgery is considered feasible and safe technique reducing the risk of catastrophic events even in high-risk cases such as the elderly, or those with comorbidities. Furthermore, it offers several advantages over conventional laparoscopic surgery, such as superior 3D vision, hinged and flexible instruments, increased range of movement, elimination of fulcrum effect, tremor free image, and ergonomic positioning for the surgeon, thereby translating to precision surgery and improved outcomes in patients [17]. Robotic surgeries have therefore been applied to various fields such as urology [18], gynecological conditions [19], and recently several thoracic surgeons have also adopted r-VATS as an option for pulmonary resections and lobectomies [10, 20–22]. One of the advantages of r-VATS over VATS is for the resection of mediastinal lesions, especially thymectomy, as the robot offers easy access to even the tight confined spaces of the anterior and posterior mediastinum [23].

**Fig. 5:**
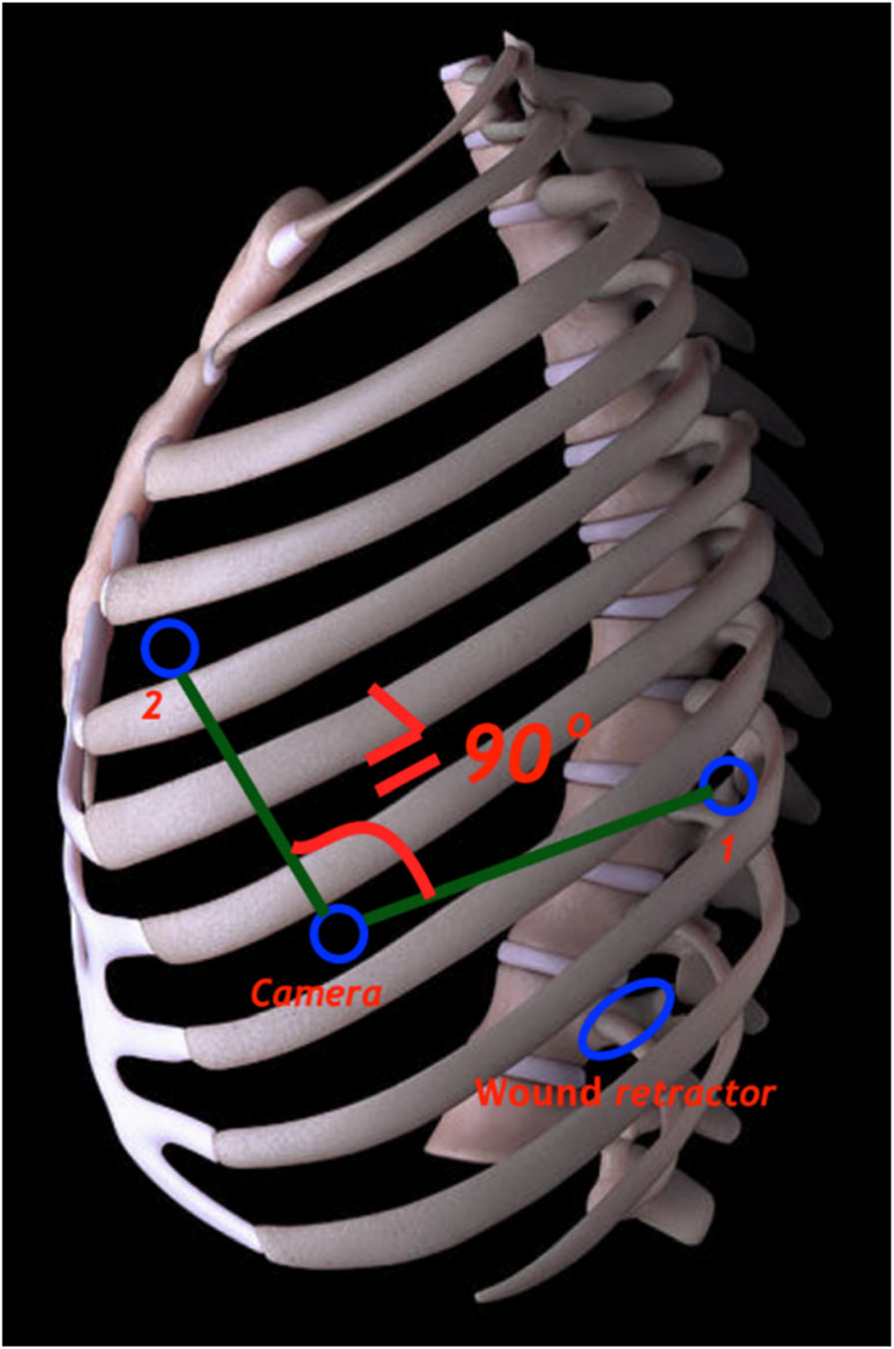
The angle created by the triangular positioning of trocars 1, the camera, and 2 was ≥90^o^. The distance between the trocars position, including the wound retractor, was more than 4 fingers wide, resulted in the robot arms to freely approach all the intrathoracic lesions easily without interfering with the assistant port. 1,2: robotic arms 1 and 2; wound retractor: 1.5 cm assistant port, acting as a working incision; camera: camera port

**Fig. 6:**
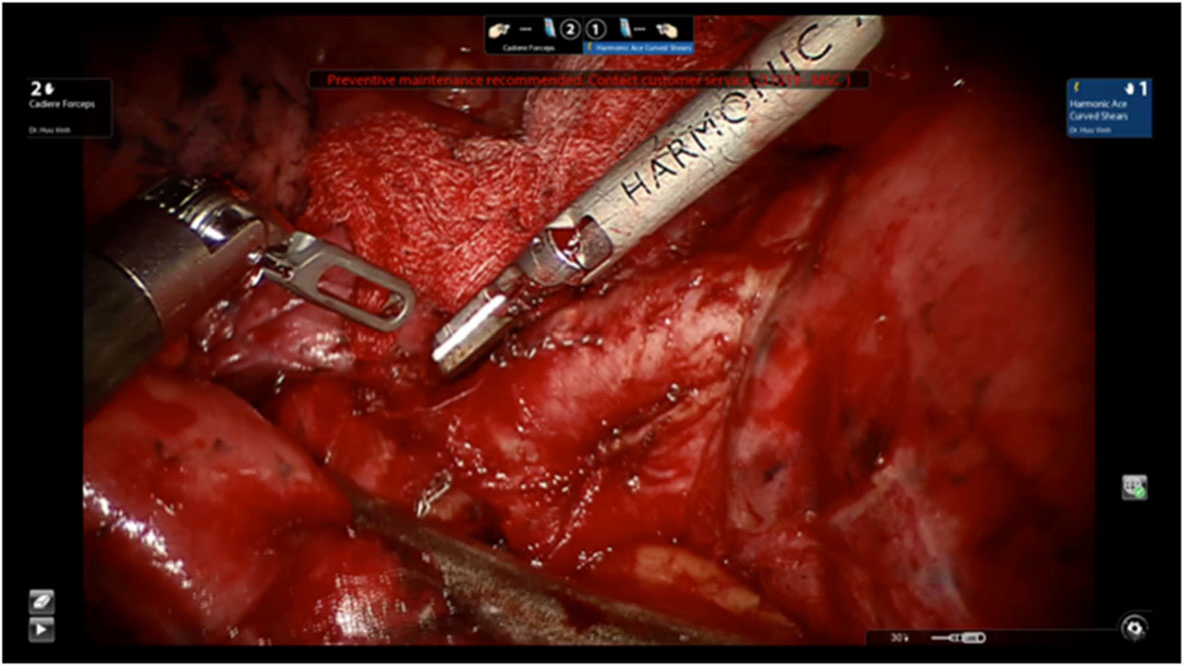
The harmonic shear with wrist was used effectively with triangular trocar concept in our r-VATS procedure, similar to that in c-VATS

All the r-VATS procedures in our study were performed using da Vinci’s Si robotic system (manufactured by Intuitive Surgicals, USA). For better access to every part of the surgical site, it is very important to place the trocars (ports) appropriately. Numerous surgeons using r-VATS across the globe and even the product manufacturer recommend placing four out of five trocars aligned in the 8th ICS, and the fifth assistant trocar at 1 or 2 lower ICS. Cerfolio et al. (2011) have described the placement of five trocars in the 7th ICS in a linear fashion as effective [23]. However, we are among the very few surgeons who have modified this trocar placement by using only 4 trocars positioned in a triangular fashion (as in c-VATS) and found it very effective (Figs. 2 and 4). Abiding by the working principle of robot arms, the angle created after the positioning of trocar 1, the camera, and trocar 2 was ≥90^o^ in our study. The distance between the position of the trocars, including the wound retractor, was more than 4 fingers wide (Fig. 5). This triangular principle provided a fast and appropriate technique for trocar placement because it allowed the robot arms to freely approach all the intrathoracic lesions easily without interfering with the assistant port. Besides, it also supported the easy use of the harmonic shear device (as used in c-VATS) in the robotic arm1 (the surgeon’s dominant hand) (Fig. 6). Today, harmonic shear is rarely used by surgeons for robotic surgeries as it is a straight device without a flexible wrist and cannot be folded like other robotic tools. Furthermore, we took advantage of the 4th port (assistant port) and used it as a working incision (just similar to that in VATS). This port, therefore, helped to serve both retracting and assisting purposes. This further eliminated the need for 5th port in our procedure, unlike in current robotic thoracoscopic surgeries. Additionally, the reduction of one port (4 instead of 5) helped to widen the distances between the ports, making it convenient for robot arms operation. In particular, it helped the assistant surgeon to be comfortable in offering supporting actions like stapling or dissection during the operation.

The triangular incision strategy has been earlier reported for r-VATS lobectomy [24], but we hereby report the application of this approach to all our robotic surgeries, including lobectomy, wedge resection, thymectomy, mediastinal tumor resection, pneumonectomy, transthoracic esophagectomy, esophageal cyst resection or esophageal diverticulum repair, and diaphragm plication. Our study demonstrated the peri- and postoperative outcomes of r-VATS in a total of 142 patients undergoing different surgical operations. Median operative time to surgery was 110 min (range = 60–280 min) and was found to be better than in previous R-VAT studies while the median length of hospital stay for our study cohort was 5 days (range 3–12 days) and was found comparable to the earlier reports [10, 25]. Conversion to open thoracic surgery was required only in 3 (2.1%) patients; 2 (1.4%) of pneumonectomy and 1 (0.70%) of mediastinal tumor resection. This may have happened because of pneumonectomy cases, generally being the toughest ones to operate. Moreover, it took us some time to familiarize with the robotic procedure in the first two cases. This conversion rate is also likely to decrease as more experience is accumulated among the surgeons. No postoperative complications and death were reported in our study. These results are equivalent or comparable to prior c-VATS studies [26–28].

In lobectomy cases, we used a wound retractor for the assistant port in lateral decubitus position, eliminating the need for CO_2_ insufflation whilst relying on one lung ventilation. This provided enough room and helped us to dissect the pulmonary vessel comfortably as CO_2_ insufflation would have compressed the pulmonary vessel to a smaller size. For surgeries in the anterior mediastinum in the supine position, while the trocar for the camera was placed just below the sternum xiphoid, trocars for arm 1, 2, and assistant were put bilaterally. CO_2_ insufflation by two- lung ventilation process was used in these cases, and this approach was found to provide more space in the anterior mediastinum as compared to semi-lateral, one-lung ventilation approach. Similarly, in some of our earlier cases, we used semi-lateral one-lung ventilation access but soon switched to a subxiphoid approach [29, 30].

Robotic surgeries such as r-VATS may be far superior to c-VATS performed by humans, but similarly, like humans, robotic surgical systems may be made to work very well with instruments without wrist (such as the harmonic shear). Nowadays, many thoracic surgeons are keen to learn robotic systems and use them for their surgeries. This shift in learning new robotic techniques should be encouraged and supplemented by adequate training in this field. The transition from c-VATS to r-VATS using robots would be a better option for those surgeons who already have good experience in c-VATS background and techniques.

## Conclusion

In summary, this study has shown that r-VATS is safe, effective, and a good alternative to c-VATS for different types of operations. All our patients were successfully managed with our modified r-VATS procedure without any operative mortality. The strategy of placing three trocars in a triangular manner and a working incision (total four ports), as in c-VATS could be well applied to r-VATS. Our team of surgeons and all patients were fully satisfied with operative outcomes; however, establishing the superiority of r-VATS using our approach, over c-VATS needs further investigation.

## Data Availability

Not applicable.

## Abbreviations

VATS: Video-assisted thoracoscopic surgery
r-VATS: Robotic video-assisted thoracoscopic surgery
c-VATS: Conventional VATS
RATS: Robotic thoracoscopic surgery
AESOP: Automated Endoscopic System for Optimal Positioning
